# Supplementary material for: Greening of the Sahara suppressed ENSO activity during the mid-Holocene
Source: Nat Commun. 2017 Jul 7;8:16020. doi: 10.1038/ncomms16020 (PMC5504352; doi:10.1038/ncomms16020)
Supplement: Supplementary Information [file ncomms16020-s1.pdf]

File name: Supplementary Information
Description: Supplementary Figures, Supplementary Tables, Supplementary Notes and Supplementary References

File name: Peer Review File
Description:

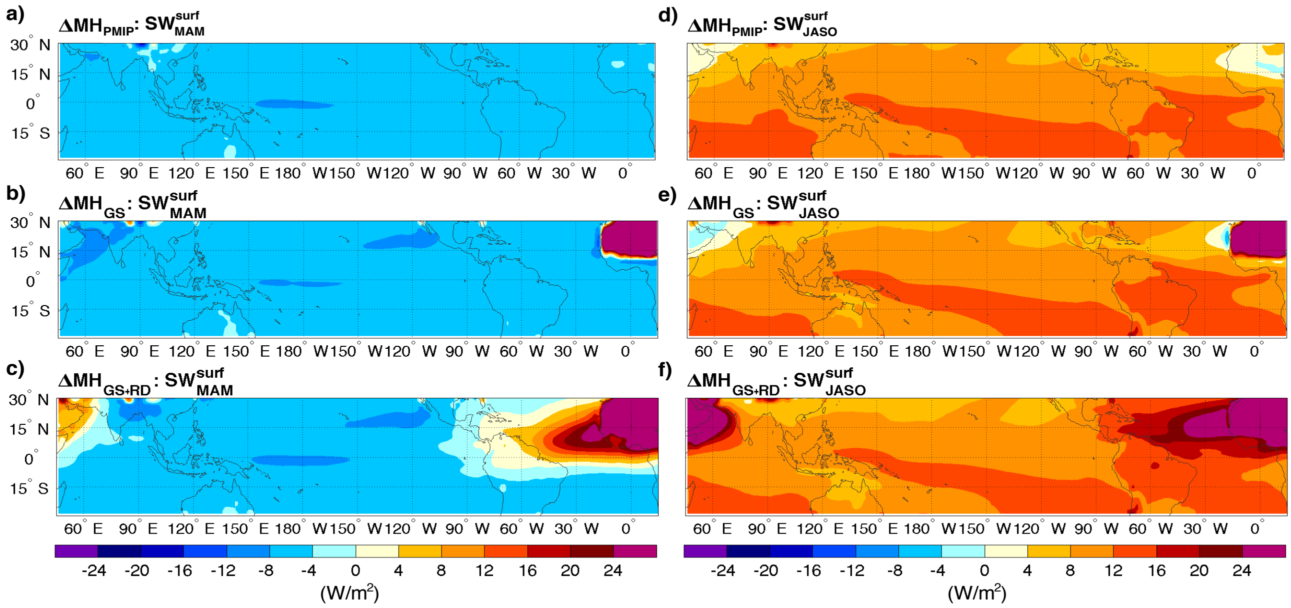

Supplementary Figure 1: Changes in tropical surface solar insolation (W/m^2) between the MH experiments and the PI simulation during the spring (MAM) and summer/early fall (JASO).

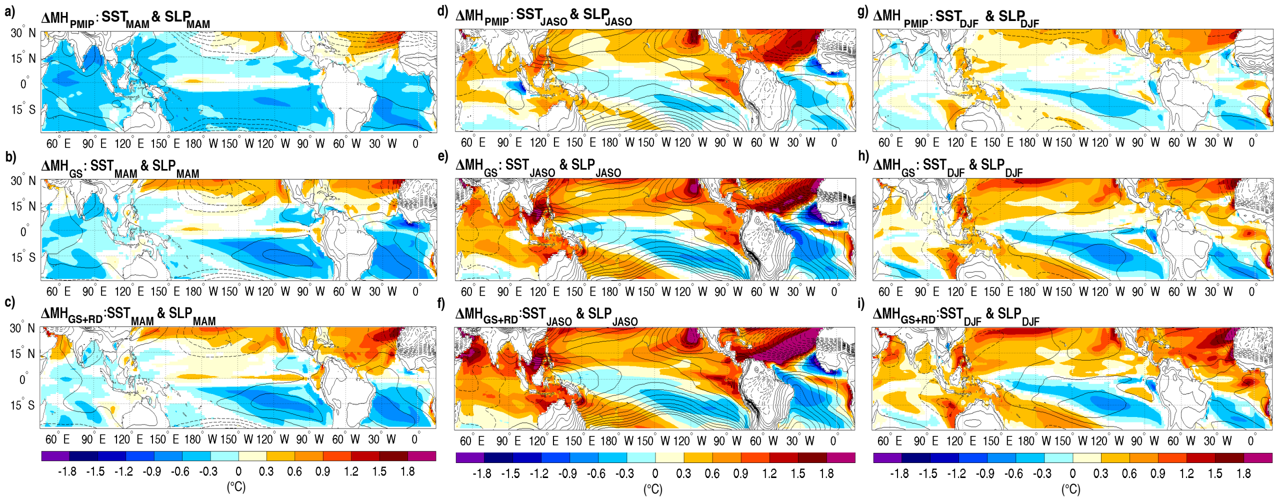

Supplementary Figure 2: Changes in sea surface temperature (SST, shadings) and sea level pressure (SLP, contours: 0.25 hPa interval from -3.5 to 3.5 hPa; 0 value omitted for clarity) for MAM (left), JASO (center) and DJF (right), and for each MH simulation relative to PI.

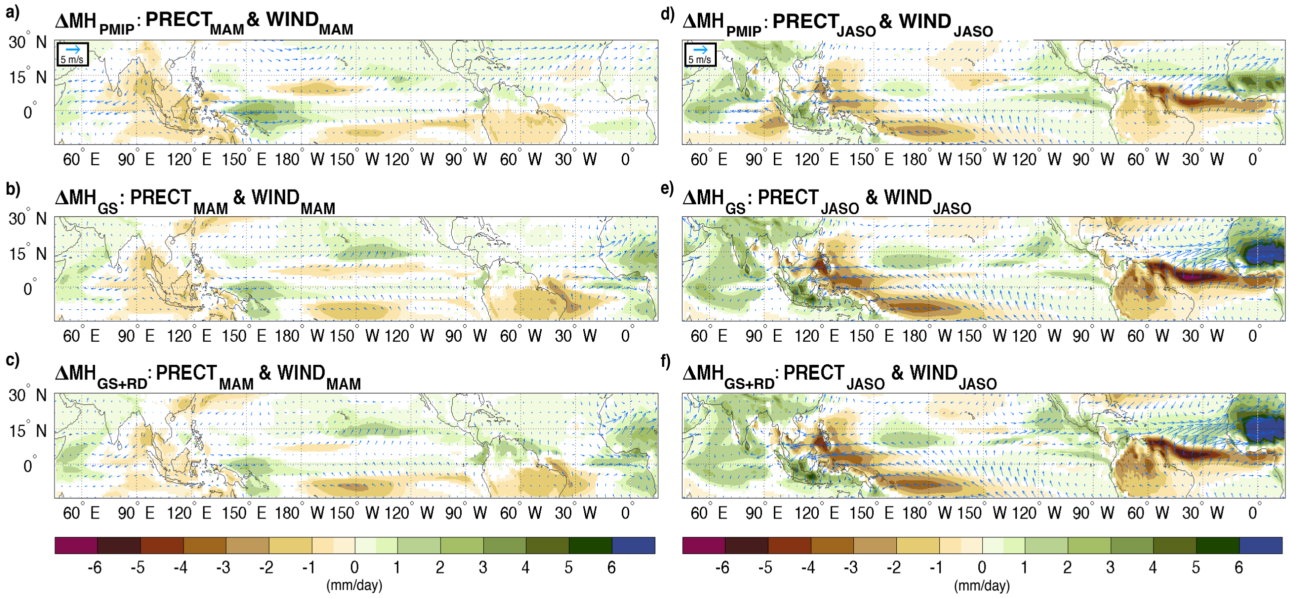

Supplementary Figure 3: Changes in precipitation (shadings) and 10 m wind (vectors) for MAM (left) and JASO (right), and for each MH simulation relative to PI.

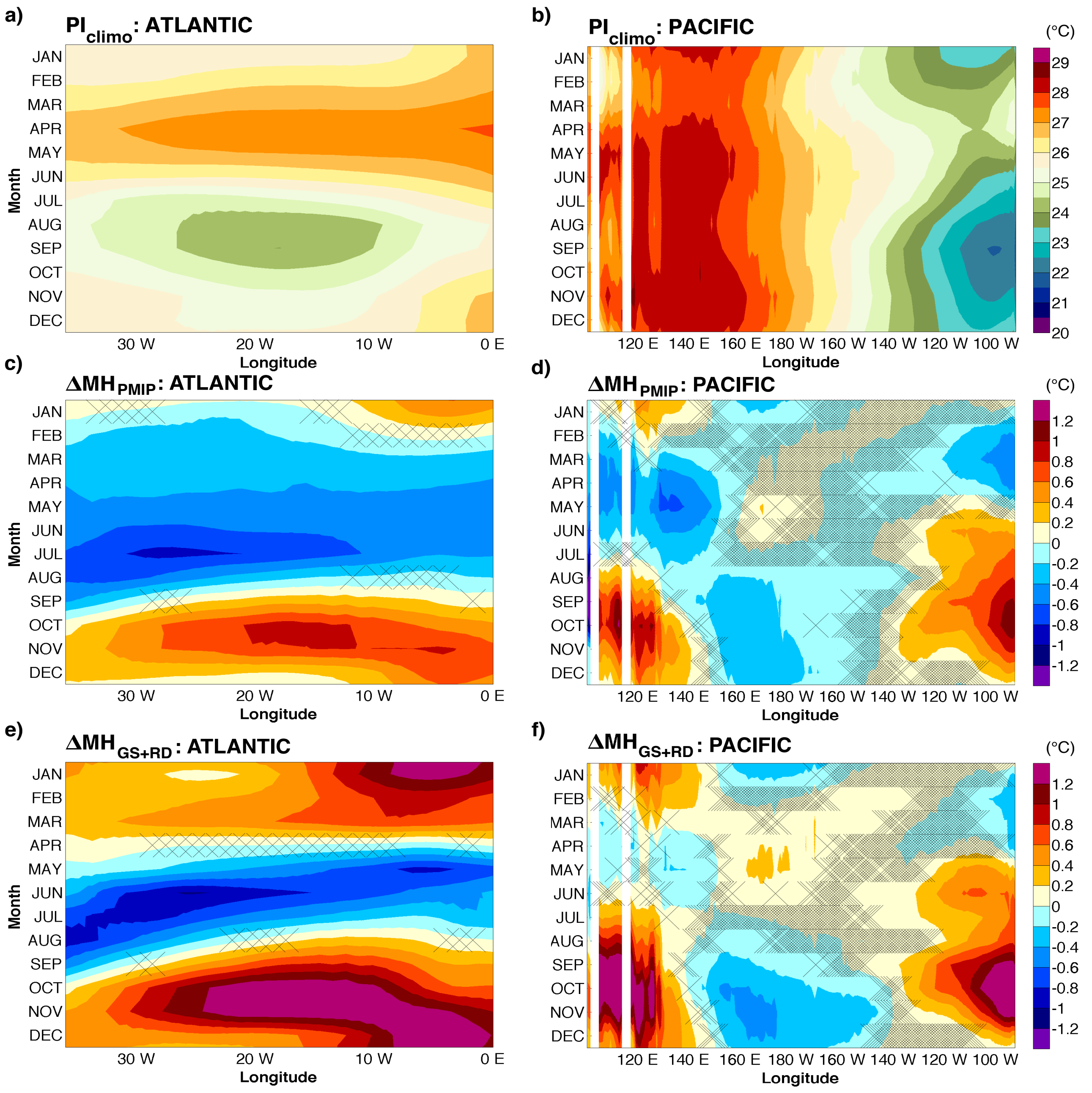

Supplementary Figure 4: a, Longitudinal transect of the climatological monthly sea surface temperature for the equatorial (3°S-3°N) Atlantic and **b**, Pacific for each month in the PI simulation and their changes in the MH (**c-d**) and MH_{GS+RD} (**e-f**) experiments. The hatched areas show regions in which the changes are not significant at 95% confidence level assessed using a two-sided *t* test.

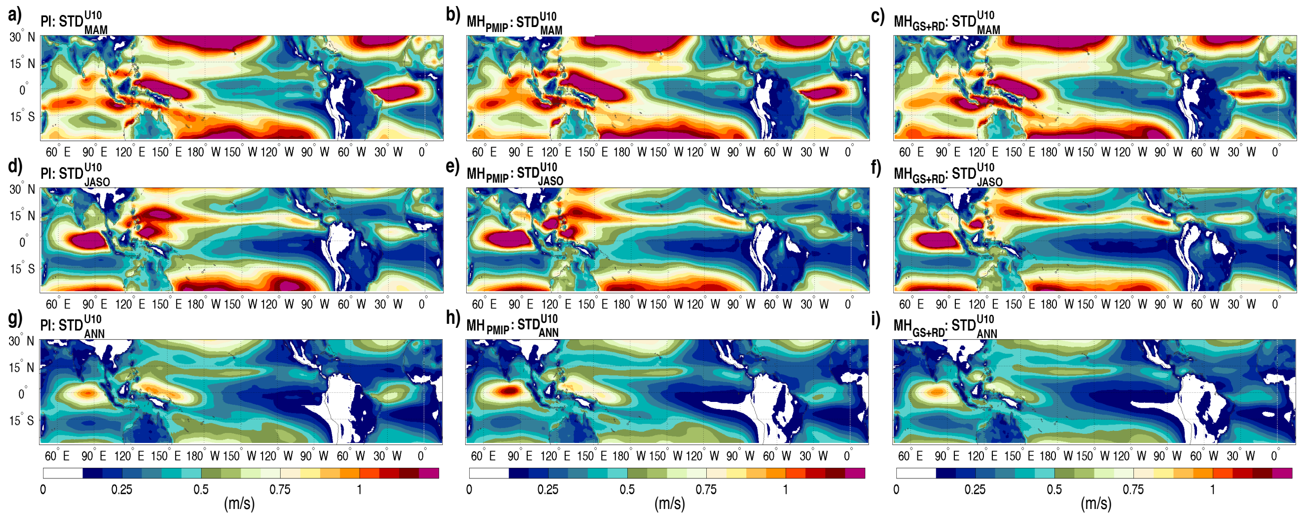

Supplementary Figure 5: MAM (left) and JASO (right) zonal wind speed at 10 m for the PI, MH and MH_{GS+RD} experiment.

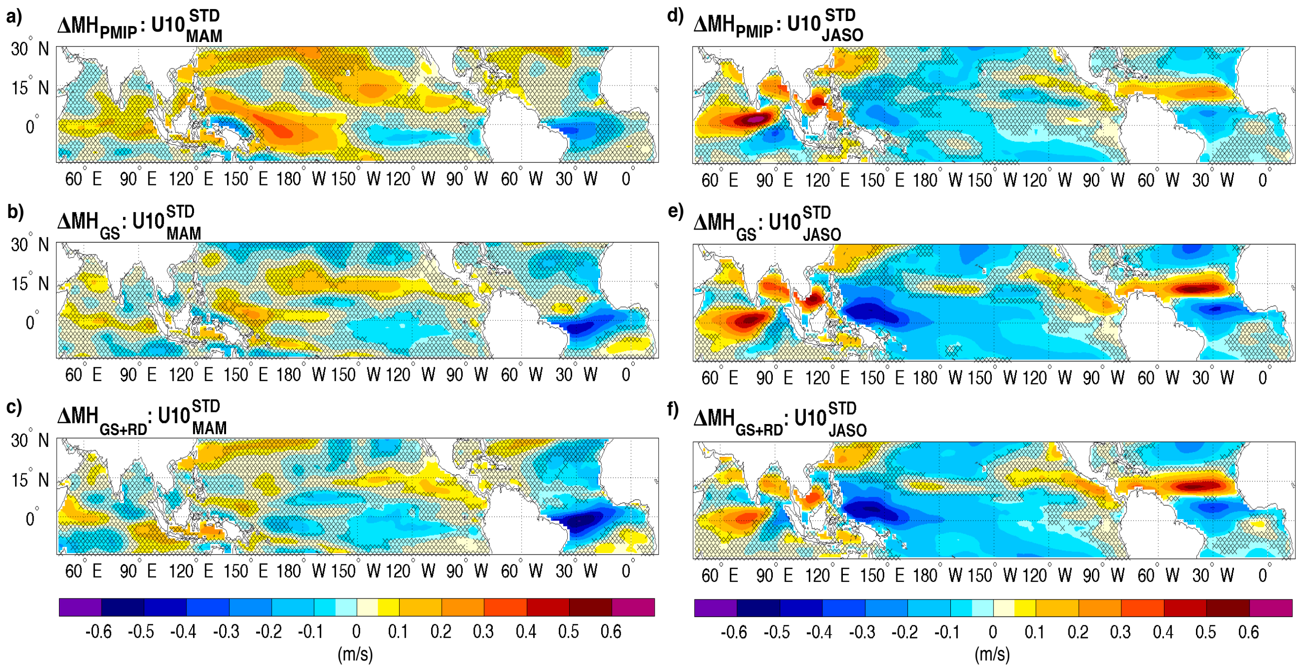

Supplementary Figure 6: Change in MAM (left) and JASO (right) 10 m zonal wind variability for each MH experiment compared to the PI.

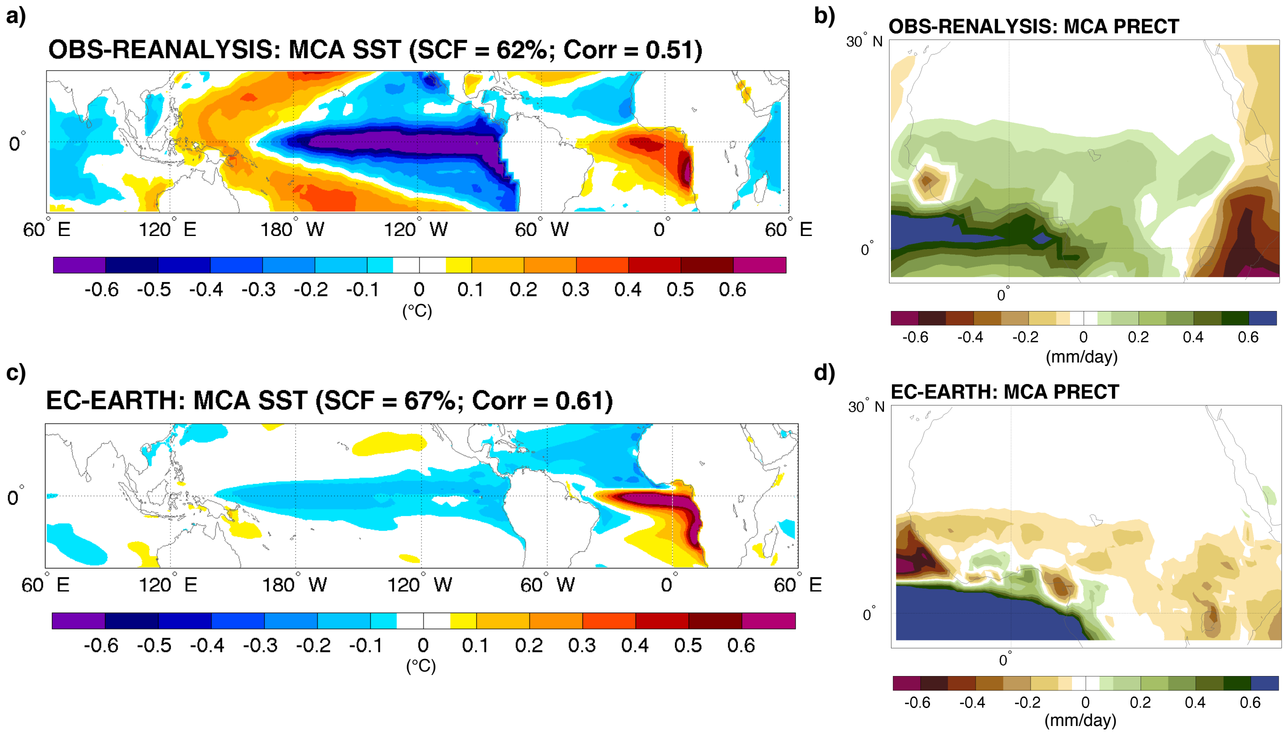

Supplementary Figure 7: (a-b) Maximum covariance analysis (MCA) of the sea surface temperature (SST) (left) and precipitation (right) and sea surface temperature (SST) (right) calculated using the observed/reanalysis GPCP Combined Precipitation Data Set Version 2.2 and HadlSST; and **(c-d)** the model data. The squared covariance fraction (SCF) and the correlation values between the SST and precipitation expansion coefficients are provided at the top of the left panels.

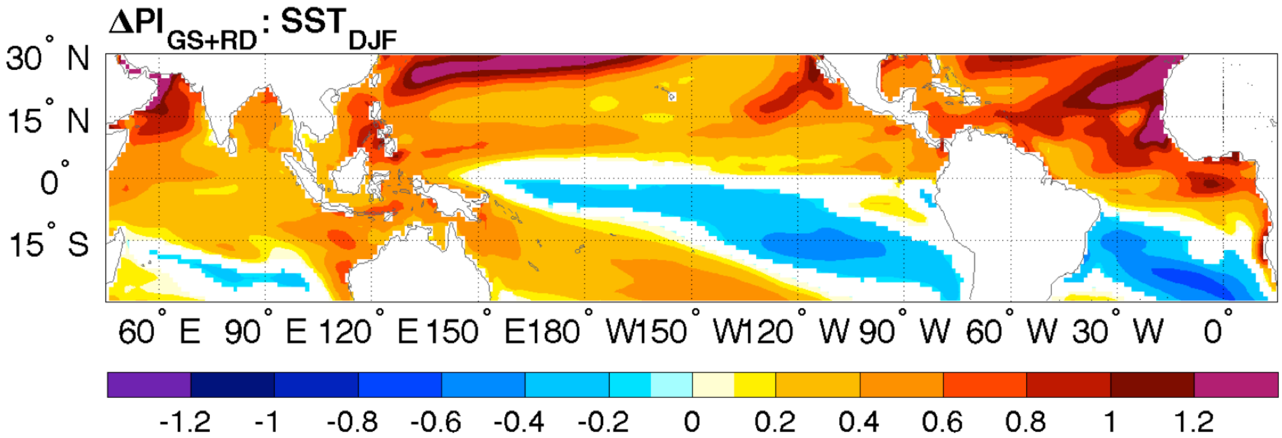

Supplementary Figure 8: Changes in JASO sea surface temperature (SST) in the PI_{GS+RD} simulation relative to PI. Only significant values at the 95% confidence level assessed using a two-sided *t* test are shaded.

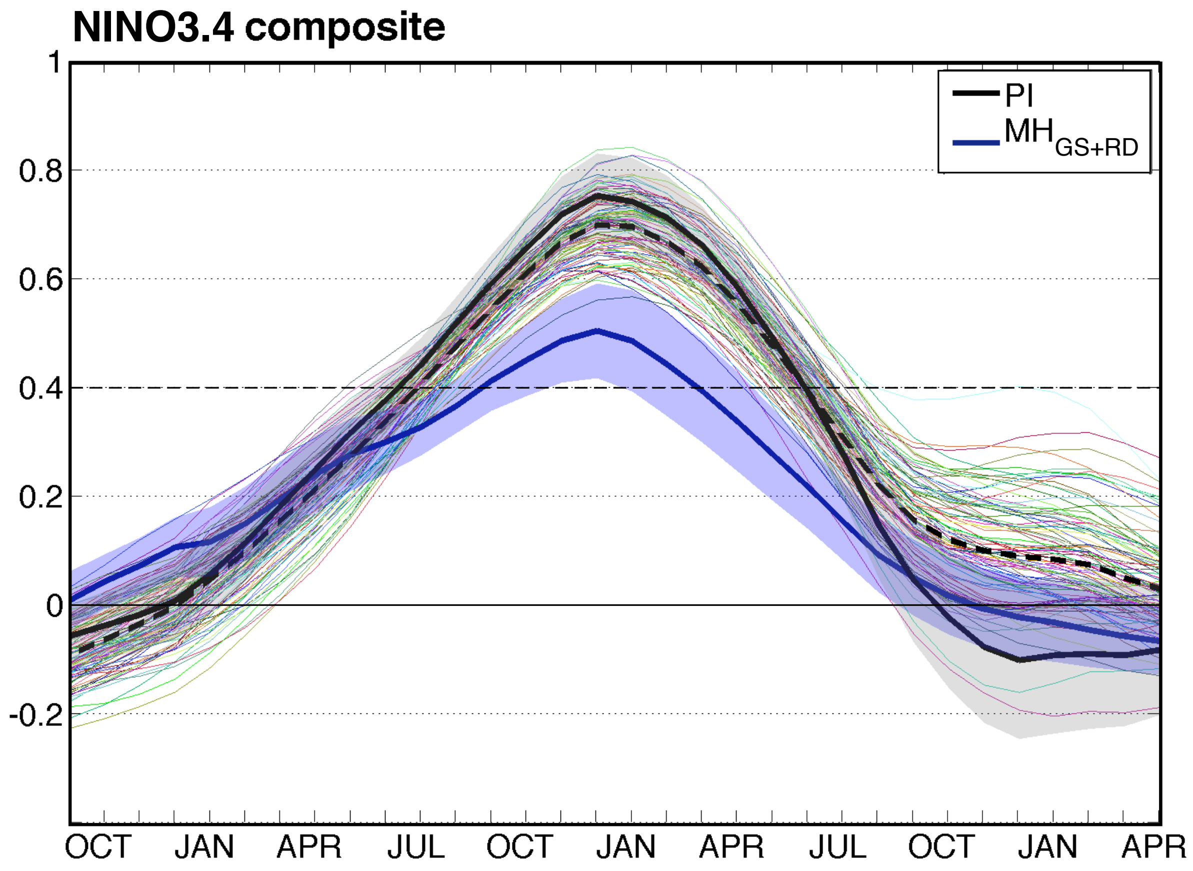

Supplementary Figure 9: Niño 3.4 index composite of the mean El Niño development and decay in the pre-industrial (PI) experiment for all 100 sub-samples, the original 200 years (in black bold, shown in Figure 3a) and the MH_{GS+RD} (in blue bold). The black dashed line show the extended 575 years of the PI simulation. The shadings show the standard error of the mean.

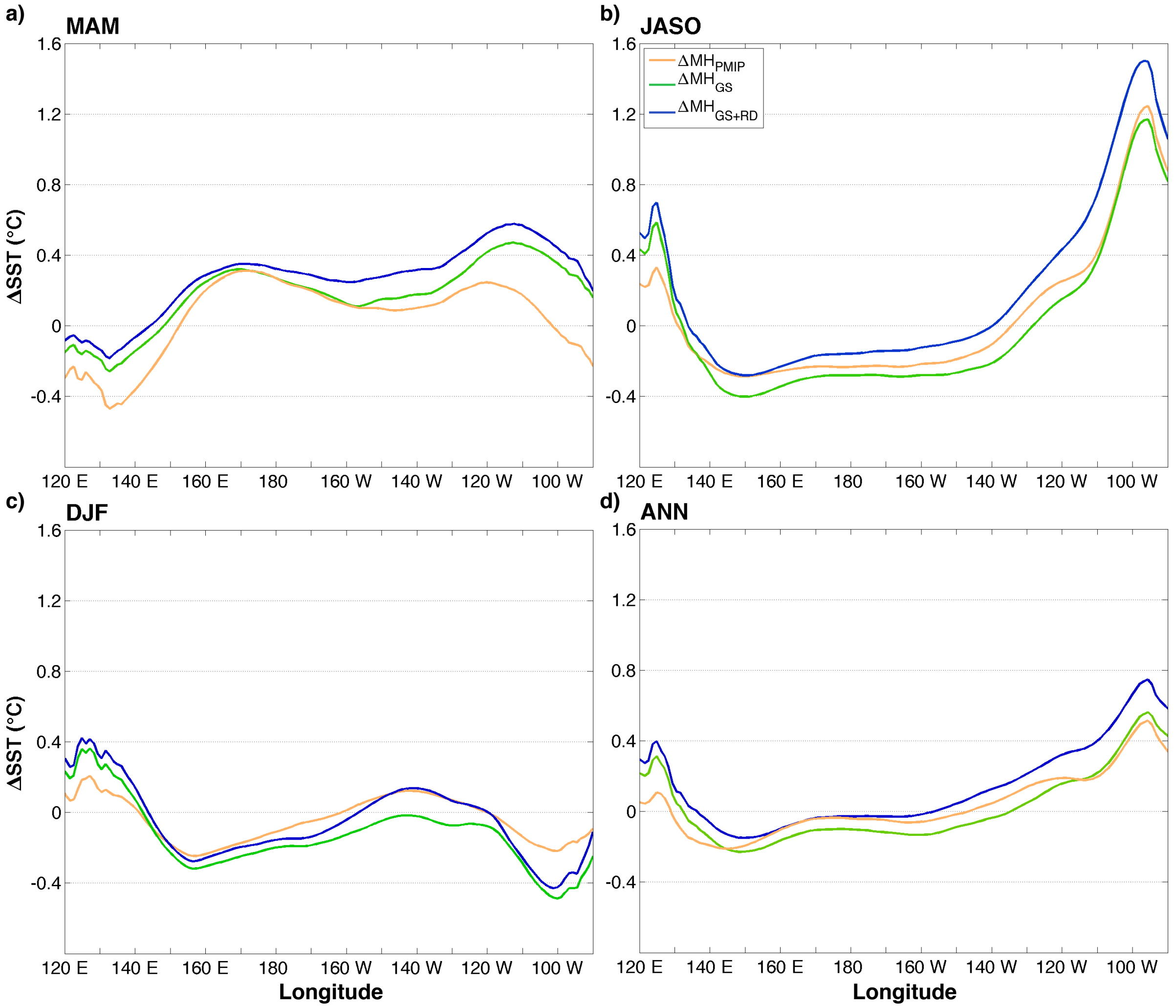

Supplementary Figure 10: Sea surface temperature changes along the equator over the Pacific for each MH experiment relative to the PI simulation.

Supplementary Table 1: Syntheses of different studies from the equatorial Pacific region used to infer the changes in temperature, precipitation and variability during early (2 – 0 ka) to mid- Holocene (6.5 – 5.5 ka).

Site	Region	Proxy	Climate parameter	MH change
Papua New Guinea[8]	Papua New Guinea	Fossil Corals	Monthly SST/monthly rainfall	Reduced variability
Papua New Guinea[9]	Papua New Guinea	Fossil Corals	Monthly SST/monthly rainfall	Reduced variability
Line Island[10] and Palmyra Atoll[11]	Central Pacific	Fossil Corals	Monthly SST	No changes
Laguna Pallcacocha[12]	Equador	Sediment gray scale	Storm events (zonal SST gradient)	Reduced variability
Laguna Pallcacocha[13]	Equador	Sediment red intensity	High SSTs (increased inorganic sediments)	Reduced variability
Galapagos Islands[14]	Eastern Pacific	Siliciclastic/ Carbonate laminae	El Niño intensity/frequency (rainfall)	Reduced variability
Core SO147-106KL[15]	Peru coast	Lithic Concentration	El Niño flood events	Reduced variability
Core 19-28[16]	Eastern Pacific	Foraminifer Mg/Ca	Annual SST	Colder SST
Core V21-30[17]	Eastern Pacific	Foraminifer Mg/Ca	Annual SST	Colder SST
Core ME0005A-24JC[18]	Eastern Pacific	Alkenones UK'37	Annual SST	Colder SST
Core KNR176-JPC32[19]	Eastern Pacific	Alkenones UK'37	Annual SST	Colder SST
Core TR163-19[20]	Eastern Pacific	Foraminifer Mg/Ca	Annual SST	No change
Archeological sites[21]	Ecuador and Peru coast	Archeological faunal assemblages	Annual SST	Warmer SST
ODP Hole 806B[20]	Western Pacific	Foraminifer Mg/Ca	Annual SST	Warmer SST
MD98-2181[22]	Western Pacific	Foraminifer Mg/Ca	Annual SST	Warmer SST
KNR176-JPC32[19]	Eastern Pacific	Alkenon δD	Annual rainfall	Wetter conditions
Gunung Buda National Park[23]	Borneo, Indopacific Warm Pool	Speleothem $\delta^{18}O$	Annual rainfall	Wetter conditions
Liang Luar cave[24]	Flores, Indopacific Warm Pool	Speleothem $\delta^{18}O$	Annual rainfall	Wetter
Core SO189-144KL[25]	Sumatra, Indopacific Warm Pool	leaf wax δD	Annual rainfall	Dryer conditions
Core BJ8-03-70GGC[26]	Sulawesi, Indopacific Warm Pool	leaf wax δD	Annual rainfall	No changes
Groote Eylandt[27]	North Australia	Pollen	Effective precipitation (summer monsoon)	Wetter conditions
Lynch's crater[28]	North-East Australia	Pollen	Surface moisture (SE-trade winds)	Wetter conditions

Supplementary Table 2: Syntheses of the different studies from the equatorial Atlantic region used to infer the changes in temperature during early (2 ka – 0) to mid- Holocene (6.5 – 5.5 ka)

Site	Region	Proxy	Climate parameter	MH change
Core GeoB4905-4 [29]	Eastern Atlantic (Cameroon coast)	Foraminifer Mg/Ca	Annual SST	Warmer SST
Core ODP-1077A [30]	Eastern Atlantic (Congo coast)	Alkenones UK'37	Annual SST	Warmer SST
Core RC24-08[31]	Central-Eastern Atlantic	Foraminifer Mg/Ca	Annual SST	No changes
Core RC24-11[31]	Central-Eastern Atlantic	Foraminifer Mg/Ca	Annual SST	Colder SST
RC13-189	Central-Western Atlantic	Foraminifer Mg/Ca	Annual SST	Warmer SST
VM25-59	Central-Western Atlantic	Foraminifer Mg/Ca	Annual SST	Warmer SST
VM30-40	Central-Western Atlantic	Foraminifer Mg/Ca	Annual SST	Warmer SST

Supplementary Note 1

Differences in the Earth's orbit in the Mid-Holocene (MH) enhanced the amplitude of the seasonal cycle in the Northern Hemisphere and tropics. As compared to pre-industrial (PI), the insolation decreases in winter/early spring (MAM) and increases during boreal summer/early fall (JASO) (Supplementary Figure 1).

Supplementary Note 2

Changes in the mean climate

In the tropics, these changes in insolation lead to a strengthening of the monsoon systems in particular the West African Monsoon – WAM (Supplementary Figure 2).

The changes in insolation also cause a general cooling of the warmer season (MAM, Supplementary Figures 3 and 4) and a warming of the colder season (JASO, Supplementary Figures 3 and 4) in the MH relative to the PI experiment. The variation in the Earth's orbit also alters the strength of the subtropical high-pressure systems (Supplementary Figure 2) through changes in the diabatic heating associated with the monsoons[1]. As a consequence of the change in the seasonal incoming solar radiation and the strengthening of the subtropical high-pressures, the trades winds over the equatorial Pacific and Atlantic are affected, and in the Northern Hemisphere the Inter-Tropical Convergence Zone (ITCZ) is shifted northward (Supplementary Figure 2). The anomalies relative to PI simulated in the experiment in which only orbital forcing are accounted for, are overall strengthened when the greening of the Sahara is imposed (MH_{GS}) and, even more, when the dust concentrations are reduced (MH_{GS+RD}).

Supplementary Note 3

Changes in the surface wind variability

In the MH simulations, the WAM onset occurs a couple of months earlier compared to the PI simulation (Supplementary Figure 3, see also Table S2 in Pausata et al.[2]) and in the Northern Hemisphere, the ITCZ is shifted northward. These changes lead to an enhanced westerly flow over the equatorial Atlantic (Supplementary Figure 3) and decreased surface wind variability in the western part of the basin both in spring and summer (Supplementary Figures 5 and 6). The wind anomaly pattern favours a climatological Atlantic Niño mean state in the MH simulations (Supplementary Figure 4d-f). The decreased surface wind variability in the western equatorial Atlantic both in late spring and summer trigger a damping of sea surface temperature (SST) variability in the eastern side of the basin (Table 1 and Figure 2). The SST variability in JASO in the eastern Atlantic is indeed significantly correlated to the zonal surface wind variability in the western part during spring in both observation[3] (R=~0.5) and our model (R=~0.4 for the PI simulation). Such a decrease in SST variability in the equatorial Atlantic is likely to further affect the Pacific as highlighted by Martín-Rey et al.[4]. Using a coupled ocean-atmosphere model, Martín-Rey et al.[4] show that when tropical Atlantic SSTs are fixed and prescribed according to the observed monthly climatology ENSO variability is reduced compared to the case in which the SSTs are interactively simulated in both basins.

Supplementary Note 4

Changes in ENSO seasonal cycle

To investigate whether the changes in ENSO seasonal cycle in the MH_{GS+RD} experiment were significantly different compared to the PI experiment we made a Nino3.4 seasonal cycle composite with each sub-sample obtained through a bootstrap technique from the 575-year PI experiment

(Supplementary Figure 9). The analysis shows that the changes in the seasonal cycle in the experiment MH_{GS+RD} are significant relative to the PI simulation.

Supplementary References

1. Mantsis, D. F., Clement, A. C., Kirtman, B., Broccoli, A. J. & Erb, M. P. Precessional Cycles and Their Influence on the North Pacific and North Atlantic Summer Anticyclones. (2013).
2. Pausata, F. S. R., Messori, G. & Zhang, Q. Impacts of dust reduction on the northward expansion of the African monsoon during the Green Sahara period. *Earth Planet. Sci. Lett.* **434,** 298–307 (2016).
3. Nnamchi, H. C. *et al.* Thermodynamic controls of the Atlantic Niño. *Nat. Commun.* **6,** (2015).
4. Martín-Rey, M., Polo, I., Rodríguez-Fonseca, B. & Kucharski, F. Changes in the interannual variability of the tropical Pacific as a response to an equatorial Atlantic forcing. *Sci. Mar.* **76,** 105–116 (2012).
5. Leduc, G., Schneider, R., Kim, J.-H. & Lohmann, G. Holocene and Eemian sea surface temperature trends as revealed by alkenone and Mg/Ca paleothermometry. *Quat. Sci. Rev.* **29,** 989–1004 (2010).
6. Timmermann, A., Sachs, J. & Timm, O. E. Assessing divergent SST behavior during the last 21 ka derived from alkenones and G. ruber -Mg/Ca in the equatorial Pacific. *Paleoceanography* **29,** 680–696 (2014).
7. Liu, Z. *et al.* The Holocene temperature conundrum. *Proc. Natl. Acad. Sci. U. S. A.* **111,** E3501–5 (2014).
8. Tudhope, A. W. *et al.* Variability in the El Niño-Southern Oscillation through a glacial-interglacial cycle. *Science* **291,** 1511–7 (2001).
9. McGregor, H. V. & Gagan, M. K. Western Pacific coral δ18O records of anomalous Holocene variability in the El Niño-Southern Oscillation. *Geophys. Res. Lett.* **31,** L11204 (2004).
10. Cobb, K. M. *et al.* Highly variable El Niño-Southern Oscillation throughout the Holocene. *Science* **339,** 67–70 (2013).
11. Cobb, K. M., Charles, C. D., Cheng, H. & Edwards, R. L. El Niño/Southern Oscillation and tropical Pacific climate during the last millennium. *Nature* **424,** 271–276 (2003).
12. Rodbell, D. T. An 15,000-Year Record of El Niño-Driven Alluviation in Southwestern Ecuador. *Science (80-.).* **283,** 516–520 (1999).
13. Moy, C. M., Seltzer, G. O., Rodbell, D. T. & Anderson, D. M. Variability of El Niño/Southern Oscillation activity at millennial timescales during the Holocene epoch. *Nature* **420,** 162–5 (2002).
14. Riedinger, M. A., Steinitz-Kannan, M., Last, W. M. & Brenner, M. A ~6100 14C yr record of El Niño activity from the Galápagos Islands. *J. Paleolimnol.* **27,** 1–7 (2002).
15. Rein, B. *et al.* El Niño variability off Peru during the last 20,000 years. *Paleoceanography* **20,** PA4026 (2005).
16. Koutavas, A., deMenocal, P. B., Olive, G. C. & Lynch-Stieglitz, J. Mid-Holocene El Niño–Southern Oscillation (ENSO) attenuation revealed by individual foraminifera in eastern tropical Pacific sediments. *Geology* **34,** 993 (2006).
17. Koutavas, A. & Joanides, S. El Niño-Southern Oscillation extrema in the Holocene and Last Glacial Maximum. *Paleoceanography* **27,** PA4210 (2012).
18. Kienast, M. *et al.* Eastern Pacific cooling and Atlantic overturning circulation during the last deglaciation. *Nature* **443,** 846–9 (2006).
19. Pahnke, K., Sachs, J. P., Keigwin, L., Timmermann, A. & Xie, S.-P. Eastern tropical Pacific hydrologic changes during the past 27,000 years from D/H ratios in alkenones. *Paleoceanography* **22,** PA4214 (2007).
20. Lea, D. W., Pak, D. K. & Spero, H. J. Climate Impact of Late Quaternary Equatorial Pacific Sea Surface Temperature Variations. *Science (80-.).* **289,** 1719–1724 (2000).
21. Sandweiss, D. H., Richardson, J. B., Reitz, E. J., Rollins, H. B. & Maasch, K. A. Geoarchaeological Evidence from Peru for a 5000 Years B.P. Onset of El Nino. *Science (80-.).* **273,** 1531–1533 (1996).
22. Stott, L. *et al.* Decline of surface temperature and salinity in the western tropical Pacific Ocean in the Holocene epoch. *Nature* **431,** 56–9 (2004).
23. Partin, J. W., Cobb, K. M., Adkins, J. F., Clark, B. & Fernandez, D. P. Millennial-scale trends in west Pacific warm pool hydrology since the Last Glacial Maximum. *Nature* **449,** 452–5 (2007).
24. Griffiths, M. L. *et al.* Increasing Australian–Indonesian monsoon rainfall linked to early Holocene sea-level rise. *Nat. Geosci.* **2,** 636–639 (2009).
25. Niedermeyer, E. M., Sessions, A. L., Feakins, S. J. & Mohtadi, M. Hydroclimate of the western Indo-Pacific Warm Pool during the past 24,000 years. *Proc. Natl. Acad. Sci. U. S. A.* **111,** 9402–6 (2014).
26. Tierney, J. E. *et al.* The influence of Indian Ocean atmospheric circulation on Warm Pool hydroclimate during the Holocene epoch. *J. Geophys. Res. Atmos.* **117,** D19213 (2012).
27. Shulmeister, J. & Lees, B. G. Pollen evidence from tropical Australia for the onset of an ENSO-dominated climate at c. 4000 BP. *The Holocene* **5,** 10–18 (1995).
28. Turney, C. S. M. *et al.* Millennial and orbital variations of El Niño/Southern Oscillation and high-latitude climate in the last glacial period. *Nature* **428,** 306–10 (2004).
29. Weldeab, S., Schneider, R. R., Kölling, M. & Wefer, G. Holocene African droughts relate to eastern equatorial Atlantic cooling. *Geology* **33,** 981 (2005).
30. Lagerklint, I. M., Rosqvist, G., Hermelin, O. & Maasch, K. New high-resolution alkenone record of last glacial to holocene sea-surface temperature change in the east-equatorial south atlantic ocean. *Geogr. Ann. Ser. A Phys.*

Geogr. **87,** 111–124 (2005).

31. Arbuszewski, J. A., deMenocal, P. B., Cléroux, C., Bradtmiller, L. & Mix, A. Meridional shifts of the Atlantic intertropical convergence zone since the Last Glacial Maximum. *Nat. Geosci*. **6,** 959–962 (2013).
